# microRNA-27b inhibits cell proliferation and invasion in bladder cancer by targeting engrailed-2

**DOI:** 10.1042/BSR20201000

**Published:** 2021-01-07

**Authors:** Yunfei Li, Qilin Duan, Lu Gan, Wei Li, Jianggen Yang, Guixiao Huang

**Affiliations:** 1 Department of Urology, Third Affilated Hospital of Shenzhen University, Shenzhen 518000, China; 2 Clinical Medical Research Center, First Affiliated Hospital of South University of Science and Technology of China, Luohu Dsitrict, Shenzhen 518020, China

**Keywords:** bladder cancer, EN2, miR-27b, therapeutic target

## Abstract

**Background:** Bladder cancer is considered a malignant tumour characterised by great heterogeneity. Engrailed-2 may be a gene implicated in bladder cancer. Bioinformatics analysis found base pair complementation between microRNA-27b and engrailed-2. The present study aimed to investigate the reciprocal association between microRNA-27b and engrailed-2 in bladder cancer.

**Methods:** The microRNA-27b and the protein of engrailed-2 in the tissues and cells of the bladder were detected. The processes of apoptosis, proliferation, invasion, and migration of tumour cells were evaluated. The co-action between microRNA-27b and engrailed-2 was detected by a luciferase reporter system. Finally, the interaction between microRNA-27b and engrailed-2 was further verified *in vivo*.

**Results:** The study found that the expression level of microRNA-27b is lower in bladder cancer tissues and cells than that in neighbouring ordinary tissues, whereas the opposite outcome was observed regarding the expression level of engrailed-2. Furthermore, microRNA-27b expression level is not significantly linked to the age of patients with bladder cancer; however, it is significantly associated with the clinicopathological grade of bladder cancer. Notably, engrailed-2 is negatively regulated by microRNA-27b. Transfection with microRNA-27b was associated with a significant reduction in the activity of bladder cancer cells and promoted apoptosis, while engrailed-2 restoration effectively reversed the above effects of microRNA-27b on bladder cancer *in vitro* and *in vivo*.

**Conclusions:** In conclusion, engrailed-2 is engaged in the development and process of bladder cancer through the negative mediation of microRNA-27b; additionally, microRNA-27b/engrailed-2 could form a signalling pathway with a significant effect on the process of bladder cancer.

## Background

After prostate cancer, bladder cancer (BC) is the second most common malignancy of the urinary tract worldwide, with 80,470 new cases and approximately 17,670 deaths in the United States in 2019 [1]. The definite cause of BC is still unknown. Many factors have been elucidated, including carcinogens from the environment, among which the matter of smoking is viewed as the most likely disease-causing agent [1,2]. BC can be classified into two clinical categories: non-muscle-invasive BC (NMIBC) and muscle-invasive BC (MIBC) [2]. Approximately 50–70% of patients of all patients with NMIBC experience a recurrence, and approximately 10–30% of these patients may develop a highly aggressive form of MIBC with increased metastatic potential [3]. Unfortunately, there is a high risk of mortality associated with MIBC despite improvements in therapeutic strategies [4]. Therefore, it is necessary to develop new biological markers for early identification of patients with unfavourable outcomes.

The engrailed-2 (EN2) gene was identified as a homeobox-containing transcription factor. Previous reports confirmed that EN2 contributed to breast cancer as an oncogene [5]. More recently, studies have demonstrated that EN2 also plays a role in prostate cancer and BC [6,7]. Our previous study indicated that EN2 could activate the PI3K/Akt pathway while inhibiting PTEN as a substitutable cancer gene in BC, and could potentially cure BC in targeted therapies [7]. In addition, the high level of EN2 expression is related to the grade, as well as the stage, of BC in pathology. Moreover, detection of EN2 in urine may potentially be a more sensitive and specific biomarker for tracking the NMIBC protein than currently available tests [8].

MicroRNAs (miRNAs) are small non-coding RNAs that contain approximately 22 nucleotides that manage the expression of genes at the post-transcriptional level by complementarily associating with the 3′UTR of target mRNAs [9]. Many studies have shown that most tumours develop due to the dysregulation of miRNAs, which act as biomarkers for the diagnosis of disease [10]. MiRNAs also play a vital role in promoting or inhibiting cancer [11]. Similarly, researchers found that miR-27b is strongly correlated with the appearance, development, invasion, and metastasis of various tumours, thus having the potential to act as a pro- or anti-cancer factor [12–16]. Recently, a study found that excessive expression of miR-27b can significantly repress proliferation, invasion, and mobilisation of BC cells, indicating how miR-27b affects the BC suppressor gene [17].

Bioinformatics analysis found that the base pairs in complementation between miR-27b and EN2 exist, suggesting the potential ways in which the sites bind and interact with each other. Therefore, miR-27b and EN2 may interact and play a role in BC. The present study aimed to examine the interaction between miR-27b and EN2 in BC *in vitro* and *in vivo*, respectively, thereby elucidating the mechanisms responsible for the development and progression of BC.

## Methods

### Tissue samples

Paired human samples of tissues in BC and neighbouring ordinary mucosa were obtained from patients treated at the Third Affiliated Hospital of Shenzhen University (*n*=30). All cases were diagnosed as primary BC without any treatment, including chemotherapy, radiotherapy, immunotherapy, etc. The harvested tissue samples were rapidly frozen in liquid nitrogen, transferred to the environment, and stored at −80°C for further analysis. Informed consent was obtained from all participants before sample collection. This research received permission from the ethical committee of the Third Affiliated Hospital of the University of Shenzhen and was conducted according to the Helsinki Declaration.

### Cell culture and transfection

The human BC cell lines that contained RT4, T24, EJ, and normal cell line SV-HUC-1 of uroepithelium were purchased from the American Type Culture Collection (ATCC). The cells of RT4 and T24 were kept in 5A medium of McCoy (Gibco), which contained fetal bovine serum (FBS, Gibco) at a concentration of 10%, L-glutamine at concentrations of 2 mM, 100 U/ml penicillin, and 100 μg/ml streptomycin (Heclony); meanwhile EJ cells were stored in the medium of RPMI-1640 (Gibco) with the same solutions, except for 2 mM L-glutamine. The cells of SV-HUC-1 were kept in F12 medium of Ham (Gibco), supplemented with 7% FBS, L-glutamine at a concentration of 4 mM, 100 U/ml penicillin, and 100 μg/ml streptomycin (Heclony). All cells were cultivated in wet air containing 5% CO_2_ at 37°C. In terms of the process of transfection, the cells were plated on 24-well plates at 80–90% confluence. Constructed plasmids were transfected into cells using Lipofectamine 2000 (Invitrogen) according to the manufacturer’s instructions. The efficiency of the transfection process was detected by qRT-PCR or WB.

### Quantitative real-time PCR (qRT-PCR) analysis

The total RNA was abstracted from tissue samples or cells using the Trizol reagent (TAKARA) based on the instructions of the manufacturer. Reverse transcription synthesis was carried out on the cDNA with the help of the cDNA Reverse Transcription kit (Bestar™ qPCR RT kit, DBI). The qRT-PCR was conducted with Bestar™ qPCR Master Mix (DBI) on an ABI 7500 Real time PCR (ABI). Sangon Biotech (Shanghai, China) designed and synthesised primers were used. GAPDH and U6 functioned as internal suppressors to detect the EN2 and miR-27b genes, respectively. The 2^−ΔΔCt^ method calculated the cycle threshold. The sequences of all primers for qRT-PCR are listed in Table 1.

**Table 1:** The primers for qRT-PCR in the present study

ID	Sequence (5′- 3′)	Product length(bp)
GAPDH F	TGTTCGTCATGGGTGTGAAC	154
GAPDH R	ATGGCATGGACTGTGGTCAT	
EN2 F	AGAACCCGAACAAAGAGGAC	153
EN2 R	TGATCTGTGACTCGTTGAGG	
U6 F	CTCGCTTCGGCAGCACA	96
U6 R	AACGCTTCACGAATTTGCGT	
hsa-miR-27b-3p F	ACACTCCAGCTGGGTTCACAGTGGCTAAGTTC	71
hsa-miR-27b-3p R	CTCAACTGGTGTCGTGGAGTCGGCAATTCAGTTGAGGCAGAAC	

### Western blot analysis

The lysis buffer (Genebase), including a cocktail of protease inhibitor and phosphatase inhibitors, was used to deal with the tissues and cells. The centrifugation of the lysates was used to collect total proteins at 4°C for 30 min and was quantified by BCA Kit for the detection of protein (Thermo), and then separated by SDS-PAGE through electrophoresis (Bio-Rad), followed by transferring to polyvinylidene difluoride (PVDF) membranes (Millipore). Later, the membrane was isolated with 5% milk with no fat at room temperature for one hour for the purpose of incubation with antibodies with primary features, such as anti-EN2 antibody (Santa Cruz, 1:3000, sc-293311) and anti-GAPDH antibody (Abcam, ab8245, 1:10000) at 4°C for the whole night. The next day, the HRP-conjugated Goat anti-Rabbit IgG was used to incubate the membrane in the role of the secondary antibodies (BOSTER, BA1054, 1:20000) for 40 min at room temperature. ECL Plus Western Blotting can be used to identify the target proteins (Millipore), while GAPDH was used to quantify the level of protein serving as a control.

### Plasmid construction

Overexpressed EN2 plasmids were constructed using the pcDNA3.1 vector. Briefly, the recombinant pcDNA-EN2 or pcDNA-EN2-NC (named as EN2-OE or EN2-NC) was placed into *Escherichia coli*, in which the positive clones were selected for amplification. Among the clones with positive features, the recombined plasmids were refined and examined using analytical methods from restriction and sequence analysis (Sangon Biotech), which was used to transfect into the cells of EJ with Lipofectamine2000 (Invitrogen). Simultaneously, plasmids containing EN2-NC were constructed and transfected into EJ cells as a control.

### Dual luciferase reporter assay

In short, the wild-type (WT) of EN2 3′-UTR, including the assumed site of combination of miR-27b-3p and EN2 3′-UTR mutant type (MT), was added into the psiCHECK-2 vector (Promega), respectively. The vector was used to transfect together with miR-27b-3p mimics into EJ cells using Lipofectamine 2000 (Invitrogen). According to the sequences of the EN2 3′-UTR and psiCHECK-2 vector, the primers for PCR were designed as follows: F: 5′-CCGCTCGAGGCTCCATTATATGACATTGGACAC-3′, R: 5′-ATTTGCGGCCGCGGTACGCTGGGCTGCTTCC-3′, and the length of the amplified fragment was 318 bp. The system for the analysis of the double luciferase reporter (Promega) was used to detect the luciferase activity of the EN2 3′-UTR according to the manufacturer’s instructions.

### Detection of cell proliferation by the CCK-8 assay

In the present study, Cell Counting Assay Kit-8 (CCK-8, Sigma) was used to detect cell proliferation. Transfected EJ cells were cultured in 96-well plates at a density of 1 × 10^4^ cells/well. The cells were treated with the reagent CCK-8 at a concentration of 10 μl/well at 37°C for 2 h when they were cultured at 24, 48, and 72 h, respectively. Finally, a microplate reader was used to measure the degree of absorbance at 450 nm (Bio-Rad).

### Cell migration and invasion assays

The 24-well plates were used to determine the migration and invasion processes of the cells. Briefly, 1 × 10^4^ transfected cells of EJ were put into the upper chambers of the transwell inserts in the medium with no serum containing 0.2% BSA, and the lower chambers were filled with medium with 10% FBS serving the function of a chemoattractant. After incubating for 48 h at 37°C with 5% CO_2_, 4% formaldehyde (Sigma) was used for 20 min at room temperature to fix the cells that had invaded the membrane. Thereafter, the cells were stained with Crystal Violet at a concentration of 0.1% (Sigma). The stained cells were imaged and counted under a microscope (Olympus CX41). In order to determine the invasion of cells, Matrigel matrix (BD Bioscience) was used to coat the upper chamber of the transwell inserts in advance, while the remaining procedures were similar to the protocols used for cell migration. Both the cells that had migrated and invaded the membrane were counted and used in three independent experiments.

### Detection of cell apoptosis by flow cytometry

Briefly, cold phosphate-buffered saline solution (PBS) was used to wash the collected cells twice in order to remove the floating cells before they were marked with Annexin V-FITC (BD Biosciences, San Jose, CA, U.S.A.). FlowJo 10.0 software was used to analyse the data.

### Animals and xenograft

In this research, both the programs and experiments involving animals conformed to the norms of organisations like the Health Guide for Care and Use of Animals in Laboratory in National Institutes. The study was approved by the Animal Care and Use Committee of the Third Affiliated Hospital of Shenzhen University. The animals used in the present study were properly cared for and the experiments were performed in accordance with the Animal Welfare Act and the ‘Guide for the Care and Use of Laboratory Animals’ from the Institute of Laboratory Animal Resources. Nude male mice aged 6–8 weeks [BALB/c A-nu (nu/nu)] were bought from SLAC Laboratory Animal Co. Ltd., Shanghai, China, and nurtured in the Animal Resource Facility in the Animal Laboratory Centre. All animal experiments were performed in SPF animal experiment center of Guangzhou Vipotion Biotechnology Co., Ltd. Cells that firmly expressed either overexpression of miR-27b-3p (miR-27b-OE), miRNA negative control (miR-NC), EN2 overexpression (EN2-OE), or EN2 negative control (EN2-NC) were injected subcutaneously into the mice in different combinations. Therefore, xenografted animals were divided into four groups: the miR-27b-OE+EN2-OE group, the miR-NC+EN2-OE group, the miR-27b-OE+EN2-NC group, and the miR-NC+EN2-NC group. The tumour volume was checked and recorded every 5 days for 40 days. On the 40th day, the mice were killed by sodium pentobarbital (10 mg/kg intraperitoneal body weight) with the preliminary tumours and organs being harvested. The paraformaldehyde was used at a concentration of 4% to fix the tissues that were kept in paraffin for further study.

### Immunohistochemistry (IHC)

Sections of tissues with four micrometres fixed in formalin and kept in paraffin were cut and placed in the silanized glass slides. Xylene and graded ethanol were then deparaffinized, followed by rehydration of the slides in graded ethanol at concentrations of 100%, 95%, 70%, and 50% in proper sequence. Ultimately, the treated slides were immersed in PBS. The solution of hydrogen peroxide at a concentration of 3% was used to isolate these sections in the endogenous peroxidase for 15 min. Subsequently, the sections were placed into an autoclave solution of sodium citrate at a concentration of 0.01 mol/l and at a temperature of 121°C for 3 min to retrieve the antigen. EN2 (1:500), the primary antibody, was used to incubate the sections at 4°C for the whole night and then the secondary antibody labelled by biotin was incubated at room temperature for an hour. PBS was the substitution for the primary one to conduct the negative controls, and diaminobenzidine tetrahydrochloride was used as the chromogen. Haematoxylin was then used to counterstain all sections, which were observed with a microscope suitable for observing bright fields.

### Statistical analysis

Quantitative data are shown as mean ± standard deviation (SD), and SPSS 17.0 (SPSS Inc.) was used to analyse the data using the statistical method. The mean values of the studied groups were used to make a comparison with the method of one-way analysis of variance (ANOVA). Differences between paired samples were analysed by t-test of the mated samples. *P*<0.05 was defined as the level of significance.

## Results

### Expression of miR-27b and EN2 in tissues and cells

**Figure 1:**
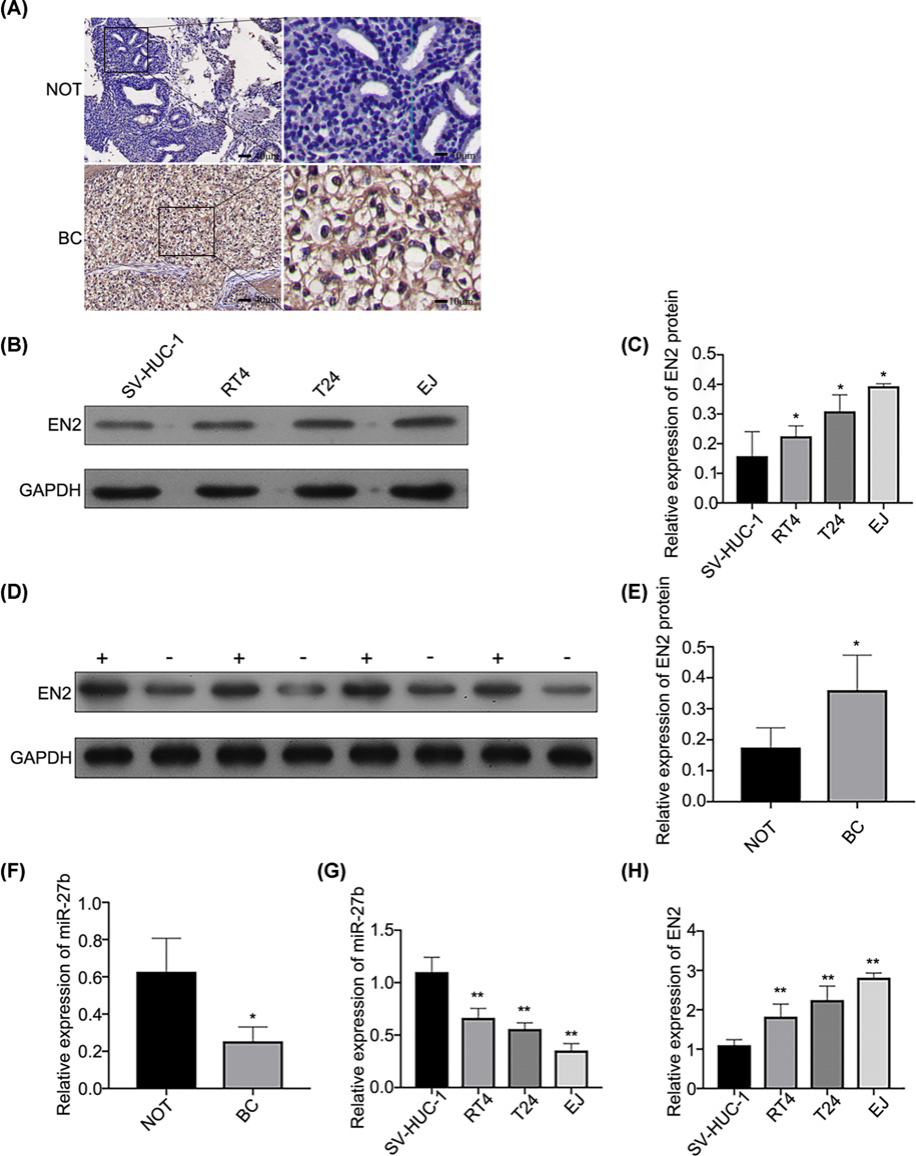
The presentation of miR-27b and EN2 expression in cells and tissues in BC (**A**) The presentation of EN2 expression in tissues of the NOT and its equivalent in BC detected by IHC (*n*=30, NOT for neighbouring ordinary tissue). (**B** and **C**) The presentation of protein of EN2 expressed in the cells of SV-HUC-1, RT4, T24, and EJ and detected by WB. *P<0.05 EJ cells versus other cells. (**D** and **E**) The presentation of EN2 expression in NOT, as well as in BC tissues, determined by WB (*n*=30; + for BC tissues; - for NOT). *P<0.05 vs. NOT. (**F**) The presentation of miR-27b expressed in the tissues of the NOT and its equivalent in BC detected by qRT-PCR (*n*=30); *P<0.05. (**G**) The presentation of miR-27b expressed in cells of SV-HUC-1, RT4, T24, and EJ determined by qRT-PCR; **P<0.01 versus SV-HUC-1 cells. (**H**) The presentation of EN2 expressed in cells of SV-HUC-1, RT4, T24, and EJ by qRT-PCR; **P<0.01 versus SV-HUC-1 cells.

The expression level of the EN2 protein in BC tissues overweighed the equivalent of the neighbouring ordinary tissues by IHC and WB, respectively (Figure 1A,D,E). In contrast, unlike the neighbouring ordinary tissues (NOT), the expression level of miR-27b in BC tissues was significantly down-regulated (Figure 1F). Further analysis found that miR-27b expression level had minimal correlation with the age of patients with BC and the clinicopathological stage of BC, despite the downwards trend observed in more advanced stages of the disease, while presenting a significant association with the pathological grade of BC (Table 2). Furthermore, in contrast with the normal SV-HUC-1 cells, miR-27b in three BC cell lines was much lower, with the lowest level in EJ cells (Figure 1G), while the expression of EN2 gene and protein in the three BC cell lines was notably higher, with the highest level in EJ cells (Figure 1B,C,H).

**Table 2:** The relationship between miR-27b expression and clinicopathological characteristics of BC patients in the present study

Clinical characteristics	*N* (case)	miR-27b (x ± s)	*P*-value
Age*			0.992
<65	12	0.2267 ± 0.08	
>65	18	0.2277 ± 0.29	
Tumor stage			0.093
Ta	8	0.2453 ± 0.07	
T1	11	0.2100 ± 0.15	
T2	6	0.1960 ± 0.15	
T3	4	0.1617 ± 0.34	
T4	1	−	
Pathological grade			0.021
Low grade	11	0.2237 ± 0.07	
High grade	19	0.1284 ± 0.23	

* 65 is defined as the cut-off age of the elderly population [18–20].

### The influence of upregulation of miR-27b-3p on the biological behaviour of BC cells

**Figure 2:**
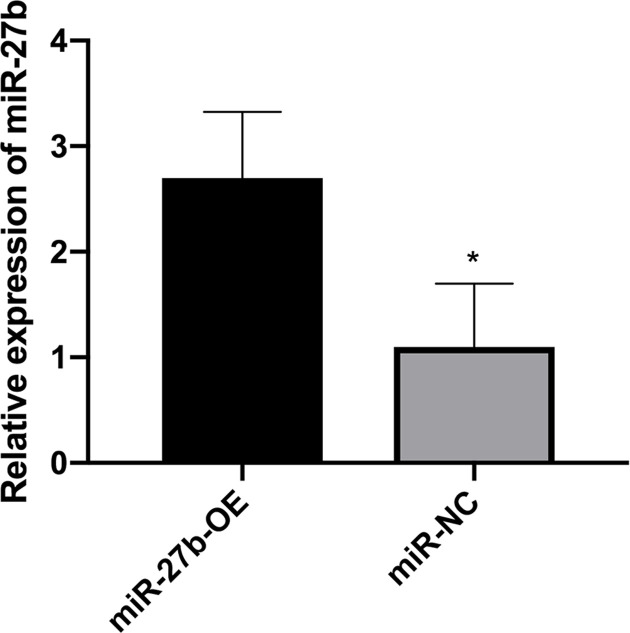
The level of expression of the miR-27b in the cell lines of EJ transfected into the miR-NC, miR-27b-OE The level of the mRNA of miR-27b increased more in miR-27b-OE than its equivalent of miR-NC (*P<0.05).

**Figure 3:**
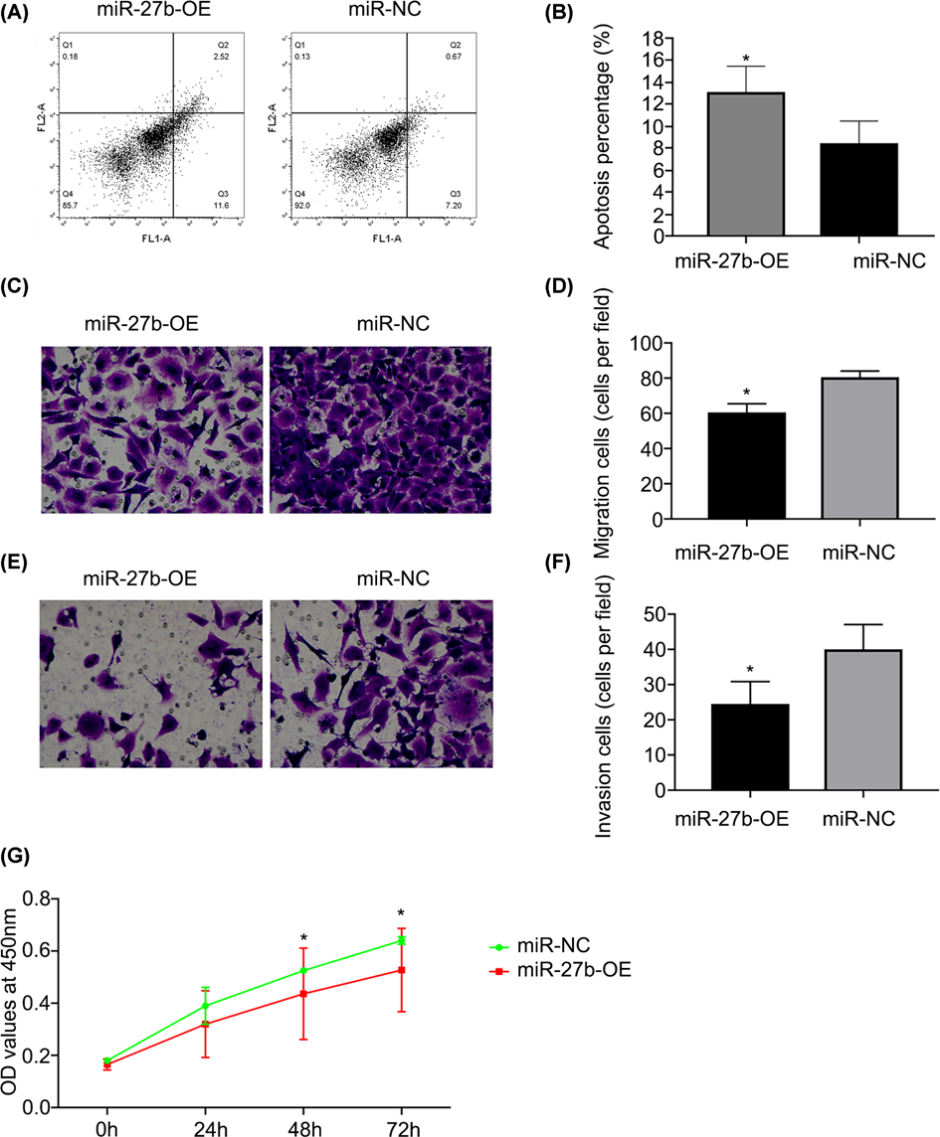
miR-27b represses BC cell proliferation, and invasion while enhancing the apoptosis of the cells (**A** and **B**) The detection of flow cytometry showed that the cells of BC reduced sharply as they were transfected by miR-27b-OE (*P<0.05). (**C** and **D**) The capacity of the cells of BC to migrate decreased with miR-27b-OE transfection (*P<0.05 vs. miR-NC). (**D**) The capacity of the cells of BC to migrate decreased with miR-27b-OE transfection (*P<0.05 vs. miR-NC). (**E** and** F**) The capacity of the cells of BC to invade decreased with miR-27b-OE transfection (*P<0.05 vs. miR-NC). (**G**) The analysis of CCK-8 demonstrated that miR-27b-OE transfection could inhibit the proliferation of BC cells 48 h later (*P<0.05 vs. miR-NC).

Next, both the miR-27b-3p mimics (miR-27b-OE) and miR-NC were used for transfection into the EJ cells, in which the collection of cells was used to make an analysis about proliferation, migration, invasion, and apoptosis. In comparison with the control, the level of miR-27b expression sharply increased after successful transfection (Figure 2). Flow cytometry analysis showed that more EJ cells underwent apoptosis after the transfection of miR-27b-3p mimics (miR-27b-OE; Figure 3A,B). Unlike the group negative control (miR-NC), the migration and invasion abilities of EJ were obviously repressed in the group with miR-27b-3p mimic (miR-27b-OE; Figure 3C–F). In addition, the CCK-8 proliferation experiment suggested that miR-27b at higher levels significantly repressed the proliferation of EJ cells, especially at 48 h after transfection of miR-27b-OE (Figure 3G). All the outcomes showed that restoration of miR-27b in BC cells could inhibit its proliferation, invasion, and migration while promoting cell apoptosis.

### miR-27b-3p inhibits EN2 by binding to the 3′-UTR of its mRNA

**Figure 4:**
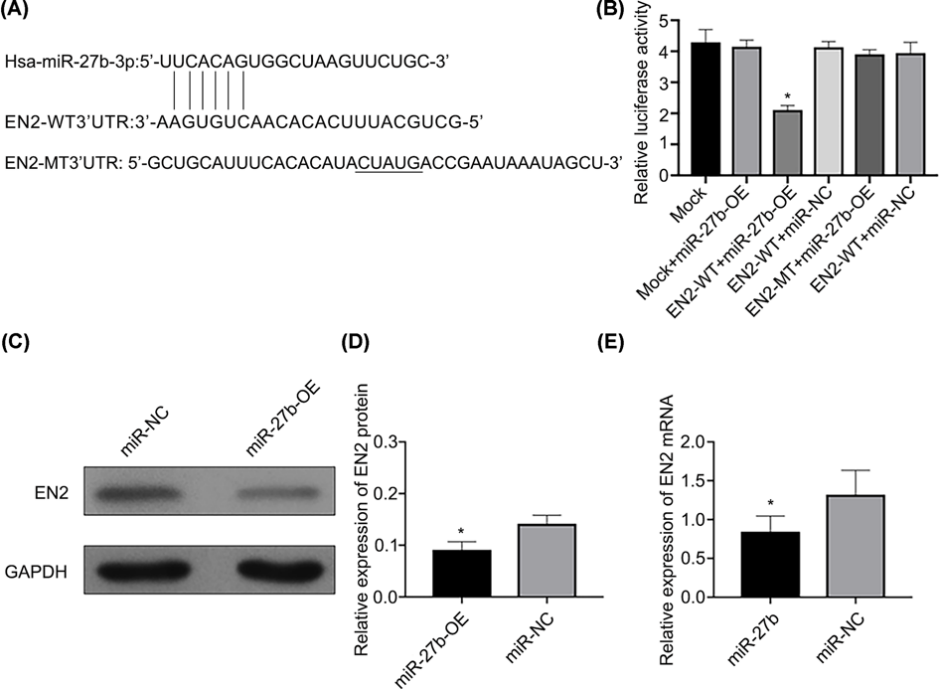
EN2 is an immediate aim of miR-27b (**A**) Sequence of miR-27b and the EN2 3′-UTR, including a combined locus of miR-27b, which can be anticipated. (**B**) The detection of luciferase in cells of BC that the miR-27b-OE and miR-NC transfected altogether, which included the EN2 3′-UTR (WT) or the mutant (MT) (*P<0.05 vs. EN2 MT). (**C** and **D**) miR-27b-OE transfection suppressed the EN2 protein levels (*P<0.05 vs. miR-NC). (**E**) miR-27b-OE transfection suppressed the EN2 gene levels (*P<0.05 vs. miR-NC).

The combined site of miR-27b-3p and the 3′-UTR of EN2 was anticipated by TargetScan and microrna.org (Figure 4A). To prove that EN2 serves as the immediate aim of miR-27b-3p, a reporter vector of luciferase was used to include EN2 wild-type (WT) or mutant 3′-UTR (MT) subcloned, and, subsequently, the process of miR-27b-3p mimics (miR-27b-OE) or the negative control (miR-NC) were used to co-transfect cells of EJ. Then, the detection of the activity of luciferase was conducted after transfection for 48 h. All the outcomes suggested that luciferase activity of the EN2-WT 3′-UTR was significantly repressed by miR-27b-3p, which had little effect on the EN2-MT 3′-UTR (Figure 4B). To test the capability of the regulation of miR-27b-3p on the expression of EN2, the transfection of miR-27b-3p mimics (miR-27b-OE) into the EJ cells was used for analysis, which showed that increased regulation of miR-27b-3p repressed the mRNA and protein levels of EN2 (Figure 4C–E).

### EN2 overexpression reverses the biological effects of miR-27b on BC cells

**Figure 5:**
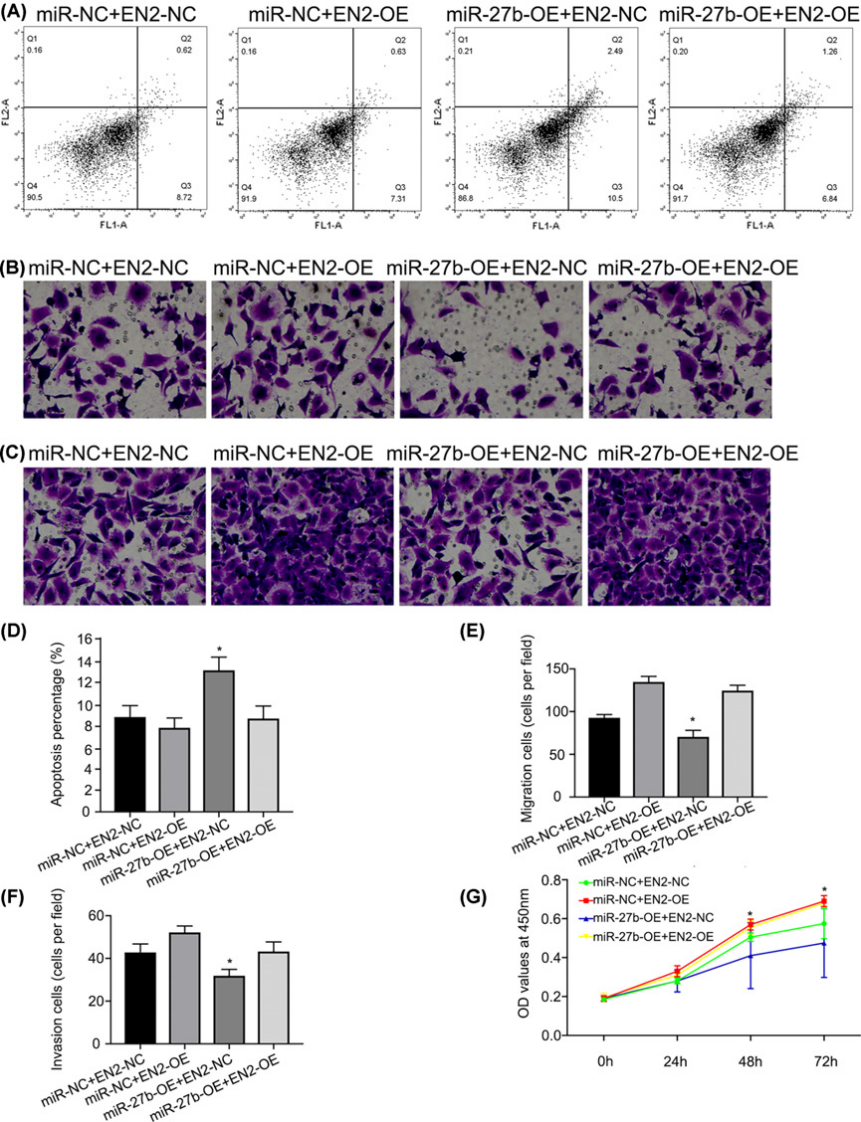
The effects of miR-27b inhibition on BC cell proliferation, invasion, and migration while promoting cell apoptosis through regulating EN2 (**A** and **D**) Flow cytometry assay revealed that miR-27b-OE transfection promoted the apoptosis of BC cells, while EN2-OE transfection reversed pro-apoptosis role of miR-27b (*P<0.05 vs. miR-NC+EN2-OE and miR-27b-OE+EN2-OE). (**B** and** F**) The invasive ability of BC cells decreased with miR-27b-OE transfection while EN2-OE transfection reversed anti-invasion role of miR-27b (*P<0.05 vs. miR-NC+EN2-OE and miR-27b-OE+EN2-OE). (**C** and **E**) The migratory ability of BC cells decreased with miR-27b-OE transfection while EN2-OE transfection reversed anti-migration role of miR-27b (*P<0.05 vs. miR-NC+EN2-OE and miR-27b-OE+EN2-OE). (**G**) CCK-8 assay showed that miR-27b-OE transfection could significantly inhibit the proliferation of BC cells, while EN2-OE transfection reversed the anti-proliferation role of miR-27b-OE 48 h later (*P<0.05 vs. the other groups).

The miR-NC, miR-27b-3p mimics (miR-27b-OE), pcDNA-EN2-NC (EN2-NC), or pcDNA-EN2 (EN2-OE) were co-transfected into EJ cell lines. At 48 h after transfection, cell apoptosis, proliferation, invasion, and migration were examined, indicating that, in contrast to the group miR-27b-OE, the recovery of EN2 contributed to the reversal of the survival actions, namely pro-apoptosis of miR-27b-3p, in which the ability of proliferation of BC cells was significantly recovered after EN2 was up-regulated (Figure 5A,D,G). Moreover, the migratory and invasive capabilities of BC cells were markedly increased by EN2 re-introduction when compared with miR-27b-3p mimics (miR-27b-OE; Figure 5B,C,E,F). In summary, the outcome showed that miR-27b-3p could repress BC cell proliferation and invasion by aiming at EN2; however, the up-regulation of EN2 could reverse the inhibitory effects of miR-27b-3p on BC cells.

### Up-regulation of miR-27b-3p suppresses BC growth, while EN2 re-introduction reversed the inhibitory role of miR-27b-3p *in vivo*

**Figure 6:**
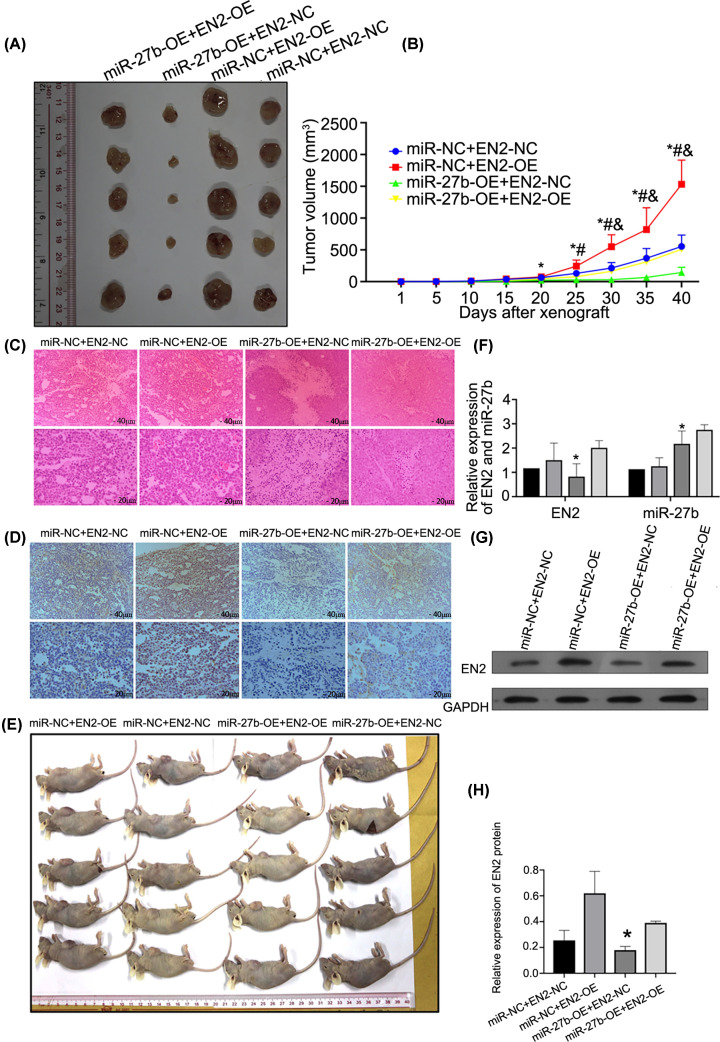
miR-27b suppressed BC growth, while EN2 re-introduction reversed the inhibitory role of miR-27b *in vivo* (**A** and **E**) Pictures of the typical mice and tumours after the mice received the inoculation for 40 days. BC cells with miR-27b-OE transfection reduced the tumour size, while EN2-OE transfection reversed the inhibitory role of miR-27b (*P<0.05 vs. the group miR-NC+EN2-OE; #*P*<0.05 vs. the group miR-NC+EN2-NC; &*P*<0.05 vs. the group miR-27b-OE+EN2-OE). (**C**) The typical pictures of xenograft tumour sections with haematoxylin stained by eosin. (**D**) The representation of protein of EN2 expressed in xenograft tumours by immunohistochemistry. The cytoplasm included most of the positive stains of EN2 and the number of cells with positive features was greater in the group miR-27b-OE+EN2-OE than in the group miR-27b-OE+EN2-NC. (**F**) The representation of miR-27b and EN2 genes expressed in transplanted tumours by qRT-PCR (*P<0.05 vs. other groups). (**G** and **H**) The representation of the protein of EN2 expressed in transplanted tumour using Western blot (*P<0.05 vs. other groups).

The nude mice were subcutaneously injected with miR-27b-3p or EN2 (*n* = 5 in each group) in order to construct the animal models. The results indicated that the volume of subcutaneous xenograft tumours in nude mice significantly decreased while miR-27b-3p was up-regulated, in contrast with the group miR-NC+EN2-NC, and a significant difference emerged at 20-day after tumour transplantation. Furthermore, EN2 re-introduction (the group miR-27b-OE+EN2-OE) recovered the growth of transplanted tumours *in vivo* (Figure 6A,B,E).

The expression of miR-27b-3p and EN2 in the transplanted tumour further verified the targeted relationship between them, as indicated by luciferase assay (Figure 6F–H). Additionally, a pathological study showed that compared with the group miR-NC+EN2-NC, miR-27b-3p up-regulation significantly alleviated histological heterogeneity of xenograft tumours and arranged the structure, hierarchy, and polar direction of cancer cells to be more regular, while EN2 re-introduction (the group miR-27b-OE+EN2-OE) reversed the above finding (Figure 6C). Immunohistochemistry indicated that compared with the group miR-NC+EN2-NC, miR-27b-3p up-regulation significantly down-regulated the expression of EN2 protein in xenograft tumours, while EN2 re-introduction (the group miR-27b-OE+EN2-OE) reversed the inhibitory effects of miR-27b-3p on EN2 (Figure 6D).

## Discussion

The present study indicated that there was down-regulation of miR-27b-3p and up-regulation of EN2 in BC tissues and cells, implicating a reciprocal action between miR-27b-3p and EN2 in the process of BC oncogenesis. A further study showed that EN2, which regulated expression, contributes to an increased level of regulation of miR-27b-3p and influences the proliferation, invasion, migration, and apoptosis of BC cells, indicating that miR-27b/EN2 could form an important signalling pathway in BC progression, particularly providing important information when treating BC and other cancers associated with miR-27b and EN2.

EN2 exerts a significant effect in many types of cancers, including breast, prostate, and epithelial ovarian cancers, in non-small cell lung cancers, and in clear-cell renal cell carcinoma [5,6,21–23]. A recent meta-analysis evaluated the accuracy of EN2 protein in urine as a biological marker in prostate cancer, indicating that the urinary EN2 presented a high specificity (89%) and low sensitivity (66%) [24]. Urinary EN2 may also be an early non-invasive diagnostic biomarker for BC. Morgan et al. found that the level of EN2 in urine was detected by ELISA with the threshold 55.5 ng/ml, and the sensitivity and specificity to diagnose BC was 82% and 75%, respectively [8]. However, it is not clear whether the path to detecting and/or screening BC EN2 deserves to advocate due to the reduced number of studies on EN2, as well as the low quality of evidence.

MicroRNAs can cause suppression of translation, and cleavage and decay of mRNA, which is led by miRNA-oriented de-adenylation of targeted mRNAs at the post-transcriptional level [25]. Substantial research has confirmed that aberrant expression of miRNAs exerts a significant effect on developing, as well as progressing, tumours, in which the miRNAs act as genes of cancer or inhibitors, serving as possible targets in therapies for various cancers [26–28].

Many studies have suggested that miR-27b influences different kinds of biological processes, such as angiogenesis, proliferation, metastasis, and resistance to drugs; therefore, it may be a promising target for the treatment of cancer [12,15,16,29]. Ishteiwy et al. reported that miR-27b was reduced in prostate cancer and that introduction of miR-23b/-27b in the lines of the cells of prostate cancer, which are metastatic and castration-resistant, was responsible for a sharp drop in the activity of Rac1 with unchanged levels, but that resulted in increased levels of the inhibitor of tumour, E-cadherin [13]. Chiyomaru et al. found that miR-27b decreased significantly in BC, and restoration of mature miR-27b largely repressed the process of migrating and invading cancer cells, indicating that the miRNA functions as an inhibitor of tumours [17].

In our research, we examined the level of expression in miR-27b and EN2 in BC tissues and cell lines. Our results indicated that unlike the tissues and cell lines of cells at the normal level, miR-27b decreased significantly in BC, which suggests that miR-27b is responsible for the pathogenesis of BC. Importantly, we found that miR-27b was significantly associated with the pathological grade of BC, while presenting a downward trend in more advanced stages of the disease, despite no notable differences due to the limited number of cases of BC in this study; this result indicates that miR-27b may have an important role in the prognosis of BC. Furthermore, overexpression of miR-27b significantly controlled the proliferation, invasion, and migration of cells while enhancing apoptosis. We anticipate that miR-27b may be used for the prognosis of BC based on clinicopathological grade and stage. Given the limited number of cases included in the study, further research is necessary to investigate the links between the expression of miR-27b and clinicopathological features of BC.

Numerous target genes existing in miR-27b can be discovered in many malignant tumours [30–32]. Wang et al. aimed to repress EN2 expression and discovered that hsa-miR-27b is up-regulated in HCMV-infected glioma cells [33]. As previously reported, the present study found that the expression of EN2 is high in BC tissues and cell lines, suggesting that miR-27b interacts with EN2 in BC. Therefore, we studied the interaction between miR-27b-3p and EN-2 functions in BC. In the present study, luciferase reporter assays demonstrated that EN2 was directly regulated by miR-27b-3p. Furthermore, transfection of miR-27b-3p significantly inhibited BC cell activity and promoted apoptosis, while EN2 restoration effectively reversed the above effects of miR-27b-3p on BC *in vitro* and *in vivo*. It was confirmed that EN2 was a direct target of miR-27b-3p.

## Conclusions

Taken together, our results increased our understanding of the effect of EN2 in the progression of BC. More importantly, the newly identified signal pathway of miR-27b/EN2, which has recently been discovered, makes it possible to study the pathogenesis of BC to improve BC therapeutic strategies. Therefore, it is necessary to perform further studies.

## Data Availability

The datasets used and/or analysed during the current study are available from the corresponding author on reasonable request.

## Abbreviations

ANOVA: analysis of variance
BC: bladder cancer
CCK-8: Cell Counting Assay Kit-8
EN2: engrailed-2
IHC: immunohistochemistry
MIBC: muscle-invasive BC
miRNA: microRNA
MT: mutant type
NC: negative control
NMIBC: non-muscle-invasive BC
NOT: neighbouring ordinary tissues
OE: overexpression
PBS: phosphate-buffered saline solution
PVDF: polyvinylidene difluoride
qRT-PCR: quantitative real-time PCR
WT: wild-type
